# Human-Like Lane Change Decision Model for Autonomous Vehicles that Considers the Risk Perception of Drivers in Mixed Traffic

**DOI:** 10.3390/s20082259

**Published:** 2020-04-16

**Authors:** Chang Wang, Qinyu Sun, Zhen Li, Hongjia Zhang

**Affiliations:** School of Automobile, Chang’an University, Xi’an 710064, Shaanxi, China; wangchang@chd.edu.cn (C.W.); lizhen@chd.edu.cn (Z.L.); zhanghongjia@chd.edu.cn (H.Z.)

**Keywords:** autonomous vehicles, lane-change decision, risk perception, mixed traffic, minimum safe deceleration

## Abstract

Determining an appropriate time to execute a lane change is a critical issue for the development of Autonomous Vehicles (AVs).However, few studies have considered the rear and the front vehicle-driver’s risk perception while developing a human-like lane-change decision model. This paper aims to develop a lane-change decision model for AVs and to identify a two level threshold that conforms to a driver’s perception of the ability to safely change lanes with a rear vehicle approaching fast. Based on the signal detection theory and extreme moment trials on a real highway, two thresholds of safe lane change were determined with consideration of risk perception of the rear and the subject vehicle drivers, respectively. The rear vehicle’s Minimum Safe Deceleration (MSD) during the lane change maneuver of the subject vehicle was selected as the lane change safety indicator, and was calculated using the proposed human-like lane-change decision model. The results showed that, compared with the driver in the front extreme moment trial, the driver in the rear extreme moment trial is more conservative during the lane change process. To meet the safety expectations of the subject and rear vehicle drivers, the primary and secondary safe thresholds were determined to be 0.85 m/s^2^ and 1.76 m/s^2^, respectively. The decision model can help make AVs safer and more polite during lane changes, as it not only improves acceptance of the intelligent driving system, but also further ensures the rear vehicle’s driver’s safety.

## 1. Introduction

Intelligent driving technologies are designed for the purpose of facilitating driving strategies that improve driving safety and reduce driver work load. Examples include, but are not limited to the: Lane-Change Decision Aid System (LCDAS), Adaptive Cruise Control (ACC), and Lane Departure Warning (LDW) [1,2,3,4]. Changing lanes is one of the most dangerous driving maneuvers, and accounts for about 5% of traffic collisions in China and Europe [5,6]. Dangerous lane change behavior is the leading cause of two-vehicle collisions, and seriously affects the safety of both the subject vehicle and rear vehicle in the target lane. Therefore, the safety of both the subject vehicle and the rear vehicle should be considered when developing Autonomous Vehicles (AVs).

Before establishing a lane changing decision strategy for AVs, it is necessary to understand the decision making process and subsequent behavior associated with lane changes. Considering how the driving environment effects driving behavior, Gipps [7] first proposed a framework governing lane-change decisions, in which the possibility, necessity, and desirability of the lane change were the main factors in determining if, when, and how the lane change was performed. Based on this decision-making framework, a lane change model was suggested by Halati et al. [8], which states that most lane change behaviors can be classified as either mandatory lane changes or discretionary lane changes; and that in general, lane-change behavior is in response to motivation, advantage, and urgency. For example, if the subject vehicle (S) cannot maintain an acceptable distance from the vehicle in front of it, S executes a lane change. Hidas [9] identified the gap between the rear vehicle (R) and S as a pivotal factor in the lane change process, and classified lane change behavior into three groups: free (discretionary lane change), forced (mandatory lane change), and cooperative. The cooperative lane change accounts for cooperation between R and S, in which R willingly decelerates, thereby positively impacting lane change feasibility. Similarly, the lane change process was described by game theory [10,11], which stated that R and S can influence each other’s driving behavior.

Nilsson et al. [12] reported that determining the appropriate time to execute a lane change is a critical issue for the development of AVs. A classic safety lane change model was proposed by Jula et al. [13], in which the Minimum Safe Spacing (MSS) (the minimum relative distance required to avoid a collision) between S and R was selected as the indicator for evaluating the feasibility of S performing a lane change. Kamal et al. [14] built on the MSS concept, and designed a lane change control algorithm for the connected vehicle. Balal et al. [15] proposed a fuzzy inference system to model lane change behavior that considers the gap between the F and surrounding vehicles. Over time, an increasing number of naturalistic driving experiments were conducted to examine the gap acceptance and obtain empirical evidence that supports that MSS theoretical research. [16,17,18]. The results showed that drivers’ gap acceptances of lane changes are basically the same.

In addition, Time to Collision (TTC) (the ratio of relative distance to relative speed between two vehicles) was extensively used in developing lane change maneuver algorithms. Lee et al. [19] used TTC to classify the lane change process into four groups, based on motivation, Lane Change Duration (LCD) (the time from the beginning of the lane change to the end of the lane change), and relative distance. Wakasugi [20] reported that the driver is able to perform a lane change when the TTC is more than six seconds. The International Organization for Standardization [21] proposed a multi-level warning model for different relative speeds of S and R, in which the lane change warning system uses TTC as the warning indicator. Bordes [22] established a similar multi-level warning model that takes into account the relative distance between S and R. While numerous studies have employed TTC as an indicator to assess lane change associated risks, there are different interpretations of the data, which have resulted in different recommendations for lane-change safety thresholds. Dijck [23] suggested that the driver may consider the lane change safe when the TTC is higher than three seconds or the gap is longer than five meters. This value is lower than the TTC finding in Hirst [24]; while Saunier and Sayed [25] suggested that the TTC can be lower than three seconds, which is lower than Dijck’s recommendation.

However, while determining a safe threshold for changing lanes, the rear and front vehicle drivers’ risk perceptions are underestimated. According to the game theory, R and S influence the driving behavior of each other. While an inappropriate lane change may directly trigger a rear-end crash between R and S, it can also stimulate a negative emotional response, like anger or anxiety, in the rear vehicle driver [26,27], who then deliberately chooses not to cooperate with F’s attempt to change lanes [28,29]. These scenarios are more dangerous for R, as they may result in R colliding with other surrounding vehicles.

There are scientists and engineers who envision that in the future, roads will be populated by mixed traffic consisting of AVs and conventional vehicles [30,31]. Many authorities are of the opinion that in order to improve trust and acceptance, AVs should mimic human-like driving behavior, which satisfies the driver’s subjective expectation [32,33,34]. Contrarily, AV driving behavior significantly benefits from being as polite as possible, since the negative impact on surrounding conventional vehicles is reduced.

To address the deficiencies in the lane-change decision model for AVs that are inconsistent with the driver’s cognition, the present work proposed a theoretical lane-change decision model with a two-level threshold by considering the different drivers’ risk perceptions and data from previous studies. Combining the MSS model with Gipps’s lane-change safety theory, the proposed lane-change decision model confirmed the Minimum SafeDeceleration (MSD) of a fast approaching rear vehicle as the safety indicator. According to the game theory, the deceleration of R, as an intuitive indicator for our model to evaluate the lane change safety, could directly relates to R’s driving behavior and willingness to cooperate. Then we implemented a naturalistic driving experiment to explore the lane change behavior and calibrate the proposed lane-change decision model. In order to acquire the discrepant safety levels of a lane change, two extreme experiments, including the front extreme moment trial and the rear extreme moment trial, were conducted, and the two-level safety threshold was determined by evaluating the risk perception of different drivers, using Signal Detection Theory (SDT). Finally, we determined the primary and secondary safe thresholds for our proposed lane-change decision model.

## 2. Related Works

The formulation of a lane-change safety indicator is the kernel of establishing a lane-change decision model for AVs, and numerous empirical studies have investigated different lane-change safety indicators. Gipps [7] first structured a lane-change decision model for urban roads that employed the requisite deceleration value of the rear vehicle in the target lane to evaluate lane-change safety. Kestinget al. [35] proposed the Minimizing Overall Braking Induced by Lane-change (MOBIL) model, and the lane-change safety criteria for discretionary lane-changes and mandatory lane-changes based on different incentives were derived. These safety criteria could reflect lane-change safety more factually on account of considering the advantages and disadvantages of other drivers. Schakelet al. [36] established an integrated Lane-change Model with Relaxation and Synchronization (LMRS) according to the naturalistic driving data. The model integrated lane-change desire and incentives with a car-following model to assess lane-change safety. The MSS model was first established by Jula et al. [13] and this model used the calculated value of the minimum longitudinal safe distance between a subject vehicle and a rear vehicle in the target lane to evaluate the safety boundary during the lane-change process. Wang et al. [37] proposed a lane-change decision model based on the Minimum Safe Deceleration (MSD) (the minimum deceleration of rear vehicle required to avoid collision) model and the deceleration of a rear vehicle in the target lane. The Lane-Change Risk Index (LCRI) model was put forward by Hyunjin et al. [38] based on the actual traffic accident data, and the model could quantize and estimate the collision risk during the lane-change process.

According to the different assessment indicators of lane-change safety, various approaches have been pursued to establish the most appropriate threshold for a lane-change decision model that can conform to the driver’s lane-change safety cognition. Gipps [7] determined the safe deceleration of rear vehicle as –4 m/s^2^, and if the calculated value was less than that threshold value, the lane-change operation was considered to be terminated. Wang et al. [37] established a lane-change decision model with a two-level threshold by calculating the deceleration of the rear-approaching vehicle, the primary and secondary thresholds were determined as 1.5 m/s^2^ and 2.7 m/s^2^, respectively. Considering that drivers with different driving styles may possess discrepancies in their cognitions of lane-change safety, Wang et al. [39] divided drivers into four different driving styles denoted as prudent drivers, lees prudent drivers, less aggressive drivers, and aggressive drivers, then different thresholds were determined for each driving style.

Currently, the TTC indicator, deduced from MSS model, has been generally applied in lane-change decision models. According to the relative speed between the rear vehicle and the subject vehicle, International Standards Organization (ISO) 17387:2008 [21] divided the TTC threshold into three different levels, and the TTC thresholds were confirmed as 2.5 s, 3.0 s, and 3.5 s, respectively, corresponding to the relative speed interval less than 10 m/s, from 10 m/s to 15 m/s, and from 15 m/s to 20 m/s. Similarly, a patent applied by BOSCH Company (Stuttgart, Germany) [22] divided TTC threshold based on different relative distance between the rear vehicle and subject vehicle, and the TTC thresholds were determined as 2.5 s, 3.0 s, and 3.5 s, respectively, corresponding to the relative distance interval from 3 m to 25 m, from 25 m to 45 m, and from 45 m to 70 m. Wakasugi [20] recommended a two-level TTC threshold of 3 s and 5 s, respectively. However, the computed TTC threshold would be easily influenced by the relative speed and distance between the rear vehicle and the subject vehicle. 

The remainder of the paper is organized as follows. Related works on lane-change decision models and safety indicator thresholds are introduced in Section 2. Section 3 introduces the naturalistic lane-change trial and extreme moment trials. Section 4 presents the lane-change decision model and the calibration parameters of the model. The two-level threshold based on the proposed model is determined in Section 5. Finally, a discussion and conclusion are presented in Section 6.

## 3. Method

On-road experiment is the main research method used in this paper, and the experiments include the naturalistic lane-change trial and extreme moment trials. The purpose of the naturalistic trial is to calibrate the parameters of the proposed lane-change decision model. The extreme moment trials are used to accurately capture the variation of driver cognition characteristics of lane-change safety. In this section, we willintroduce the required equipment, participants, test route and procedures for the experiments in detail.

### 3.1. Apparatus

**Figure 1:**
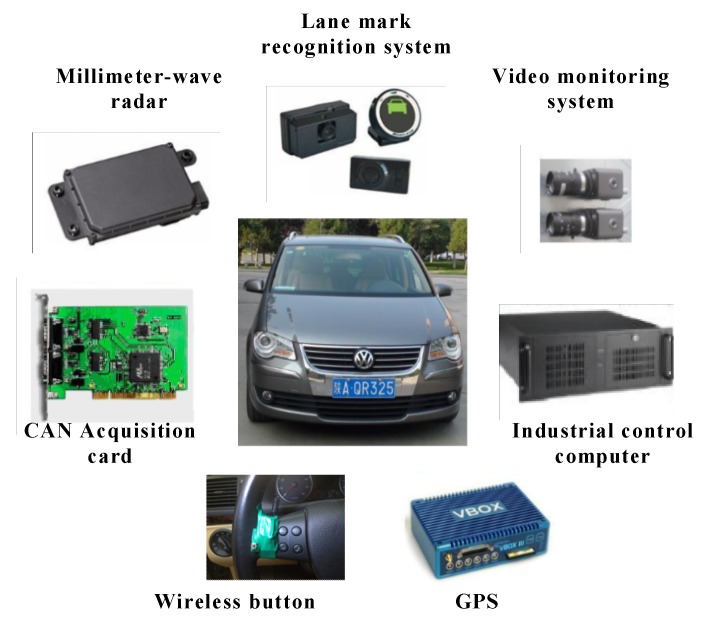
The test vehicle.

The test vehicle is depicted in Figure 1. The test vehicle used in our experiments was a 2008 Volkswagen Touran, equipped with a Lane Mark Recognition system (Mobileye C2-170, made by Mobileye Company, Jerusalem, Israel), two millimeter-wave radars for measuring the relative speed and distance between the subject vehicle (S) and the surrounding vehicles, a video monitoring system for collecting the head motion and eye movement of drivers and the driving environment, and a VBOX (a piece of equipment that can obtain vehicle’s GPS coordinate, made by Racelogic Company, London, England) to collect the driving speed and acceleration. A wireless button was fixed on the left side of the steering wheel, the button press time can be recorded.

### 3.2. Participants and Driving Route

Thirty experienced drivers participated in the two experiments. Drivers’ ages ranged from 27 to 50 years old, with an average age of 39.8 years (Standard Deviation = 7.17). Their driving experience ranged from 2 to 28 years (mean = 14.2, Standard Deviation = 8.3). All the participants were non-professional drivers with a valid driver’s license, normal or corrected vision, and experienced no traffic accidents over the past two years.

**Figure 2:**
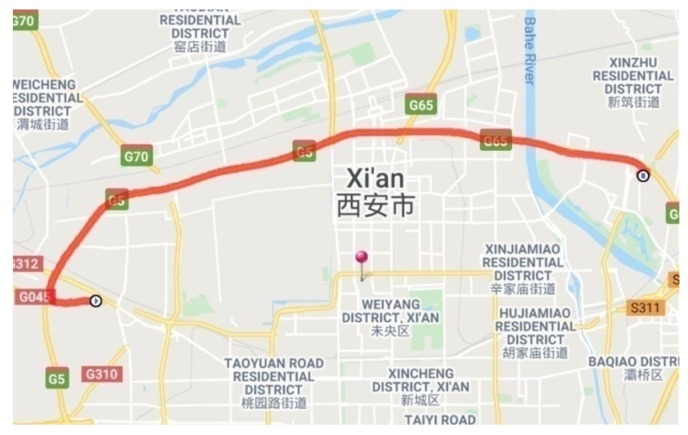
The experiment route map.

The drivers were required to drive the test vehicle on a section of highway, from Sanqiao to Xinzhu, Xi’an, China, as shown in Figure 2. The route was a 38 km, two-way six-lane road, with a 3.75 m lane width, and speed limit of 100 km/h. To keep the drivers safe while driving at a high speed, the test was carried out during non-peak hours and in clear weather conditions. To reduce driving workload and to ensure driving safety, the driving route had a zero gradient and most of highway section was straight road. Participants were paid ¥300 for their participation after they had finished all the experiments.

### 3.3. Naturalistic Lane-Change Trial

To investigate the naturalistic lane-change behavior, participants were required to drive on the test road using their own driving style, without any instructions or requirements.

To maintain driving safety, a staff member, who is an experienced driver, accompanied the driver in the front passenger seat and alerted the driver if he detected any potential risk. A second staff member was seated in the back to ensure the monitoring equipment in the test vehicle was working properly. The two staff members were instructed not to converse during the experiment except to make the driver aware of a hazardous condition or to address an equipment problem.

Data on successful lane-change maneuvers and failed lane-change maneuvers were collected. A failed lane change is when the driver intended to change lanes, but failed to perform the lane-change maneuver. A video monitoring system recorded the driver during the duration of the experiment. The intention to change lanes was detected by observing the driver’s eye and head movement, the use of turn signal lamp, and the driving environment [40,41,42,43], which was recorded by the monitoring system.

### 3.4. Extreme Moment Trials

The extreme moment trials were divided into two parts: the front extreme moment trial and the rear extreme moment trial.

The front moment is the last possible moment that the front vehicle (F), on the host lane, can safely change to the target lane without colliding with the rear vehicle (R). In the real lane-change processes, when R is driving on the target lane and is quickly approaching F, the relative distance between R and F shortens; thus, the lane-change process will gradually change from a safe to a dangerous state. In this work, the moment between the safe state and the dangerous state, i.e., the last chance for the driver to perform a safe lane change and have no negative effect on the R, is called the front extreme moment. A lane change anytime up to the front extreme moment produces no danger and the rear vehicle driver can drive normally without feeling the need to take evasive action.

Similar to the front moment, when R is approaching F quickly, there is a moment between when the rear driver feels safe and when he feels endangered. In this work, this moment is referred to as the rear extreme moment.

**Figure 3:**
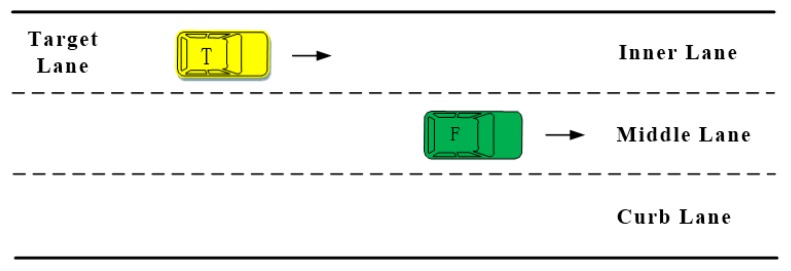
Front extreme moment trial.

Figure 3 is a schematic diagram of the front extreme moment trial. In this part of the experiment, the participants were required to drive our test vehicle (T) on the target lane, and estimate the front extreme moment. Participants were instructed to quickly approach the front vehicle, and indicate the extreme moment during their approach by pressing the wireless button.

**Figure 4:**
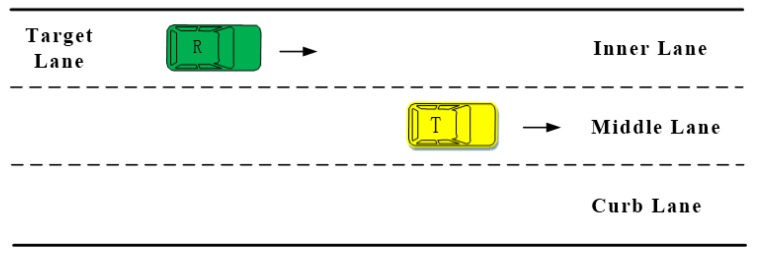
Rear extreme moment trial.

Figure 4 is a schematic diagram of the rear extreme trial. In this part of the experiment, the participants were required to drive our test vehicle (T), and estimate the rear extreme moment based on their observation of R. Participants were instructed to drive on the middle lane and to indicate the extreme moment by pressing the wireless button when R was fast approaching the test vehicle (T) from the target lane.

As with the naturalistic lane-change experiment, two staff members were seated in the front and back, respectively. During the experiment, the staff member in the back seat focused on R. When R, driving on the adjacent lane, was quickly approaching T, the staff member would ask the driver to observe R and indicate the extreme moment by pressing the button. The front staff member’s responsibility was to observe the driving environment and ensure the experiment safety. 

Due to the drivers needed to frequently observe R, the workload in this test was heavier than normal driving. To reduce the workload, the experiment was carried out in cruise control mode at speeds of 60 km/h, 70 km/h, 80 km/h, and 90 km/h.

### 3.5. Procedures

Before the experiment, the drivers were asked to participate in a practice round for approximately 10 min to familiarize them with the test vehicle and the road. Next, the participants began the naturalistic lane-change experiment. To ensure the drivers drove with their personal style, the staff member asked them to first drive along the test route and did not give them any instructions during the experiment. Following completion of the naturalistic lane-change experiment and a 10 min break, the two extreme moment experiments were carried out in a random order. Participants were given a second 10 min break between front and rear extreme moment trials.

## 4. Lane-Change Behavior Analysis

### 4.1. Lane-Change Process

To investigate the lane-change behavior, the speed of S, relative speed, distance between S and surrounding vehicles, Lane-Change Duration (LCD), Time to Line Crossing (TLC), and deceleration behavior of S were assessed.

**Figure 5:**
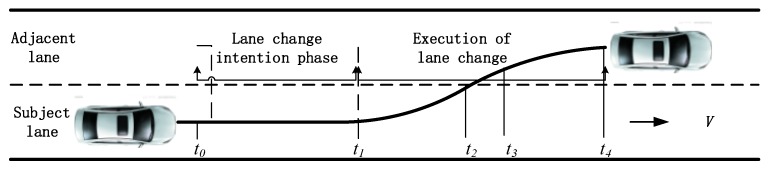
Lane-change process.

Figure 5 exhibits a complete lane-change process. *t*_0_ marks the time the driver decides to change lanes. At *t*_1_, the lane change begins (start moment); at *t*_2_, the front wheel touches the lane marking; at *t*_3_, the whole vehicle is in the adjacent lane; and at *t*_4_, the lane-change process is complete.

According to the LCD definition [44], the LCD started and ended when the vehicle began and stopped moving in a lateral direction, namely *t*_1_ to *t*_4_. Based on the TLC definition [45], the TLC started when the vehicle began moving in a lateral direction and ended when the front wheel touched the lane marker, namely *t*_1_ to *t*_2_.

### 4.2. Lane-Change Decision Model

Two safe level thresholds were proposed based on the driver’s subjective extreme moment. In this model, once it is observed that *S* started changing lanes, the lane-change model predicts the safety deceleration of R, which is the minimum deceleration to ensure that R does not collide with S.

**Figure 6:**
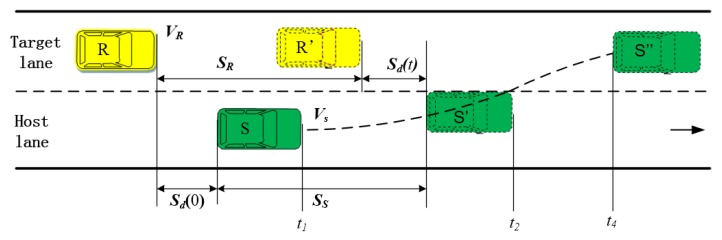
Lane change behavior.

As shown in Figure 6, in real lane-change processes, most collisions occur after S crosses the lane marking, namely after *t*_2_. Thus, *t*_2_ to *t*_4_ is the high-risk traffic conflict period during the lane-change process. Therefore, the lane-change safety evaluation should be completed before *t*_2_ at the latest to effectively reduce the accident rate.

To establish an intuitive lane-change decision model, the MSD of R during the lane change of S was selected as the indictor, which ensures R does not collide with S. This proposed model asserts that lane changes can be safely performed when the MSD is lower than a specific safe threshold. In some safety distance models, the safety distance was calculated according to the vehicle’s maximum deceleration. However, on a real high-speed road, if R brakes with the maximum deceleration, the collision occurrence between R and the car behind it will increase. In reality, the driver usually does not break with maximum deceleration on a high-speed road. Therefore, in this study, two levels of safe thresholds were selected based on naturalistic driving behavior on a real highway.

The key parameters of lane-change behavior derived from the naturalistic lane-change trial include the: velocity of S (***V_S_***), velocity of R (***V_R_***), relative velocity (***V_r_***), deceleration of R, TLC, and relative longitudinal distance between S and R (***S_d_***).

Considering *t*_1_ as the initial time of the lane change, and *t*_2_ as the TLC of F, for any time before the line crossing, the longitudinal displacement of S ($S_{S}\left(t\right)$) can be calculated as:

(1) $$S_{S}\left(t\right)=V_{S}\left(t_{1}\right)t-\int_{t_{1}}^{t}\int_{t_{1}}^{t}a_{S}\left(\tau \right)d\tau dt\;t\in \left[t_{1},t_{2}\right]$$

where $a_{S}\left(\tau \right)$ is the longitudinal deceleration of S at time t after the lane-change start moment and $V_{S}\left(t_{1}\right)$ is the speed of S at *t*_1_.

Moridpour et al. [43] reported that drivers (someone who wants to change lane) keep a constant speed during the lane-change process. Therefore, Equation (1) is simplified as:

(2) $$S_{S}\left(t\right)=V_{S}\left(t_{1}\right)t\;t\in \left[t_{1},t_{2}\right]$$

The longitudinal displacement of R ($S_{R}\left(t\right)$) can be calculated as:

(3) $$\{\begin{array}{c} S_{R}\left(t\right)=V_{R}\left(t_{1}\right)t\;t\leq T\\ S_{R}\left(t\right)=V_{R}\left(t_{1}\right)t-\int_{t_{1}}^{t-T}\int_{t_{1}}^{t-T}a_{R}\left(\tau \right)d\tau dt\;t>T\; \end{array}\;t\in \left[t_{1},t_{2}\right]$$

where $a_{R}\left(\tau \right)$ is the longitudinal deceleration of R during the lane change and $V_{R}\left(t_{1}\right)$ is the speed of S at *t*_1_. Rear vehicle driver’s reaction time (*T*) is an important factor in the lane-change process. Many researchers [46,47] have investigated the reaction time during brake behavior, and found the reaction time for deceleration is about 1 s.

In Equation (3) describes the total longitudinal displacement of R from *t*_1_ to *t*.*t* ≤ *T* means the driver of R has not yet responded, and R continues to drive at a constant speed of $V_{R}\left(t_{1}\right)$. During this period, the total longitudinal displacement of R is $V_{R}\left(t_{1}\right)*t$.*t* > *T* means that the driver of R has responded to the braking of S, R maintains a deceleration of $a_{R}\left(\tau \right)$. During this period, the total longitudinal displacement of R from t_1_ to t is $S_{R}\left(t\right)=V_{R}\left(t_{1}\right)t-\int_{t_{1}}^{t-T}\int_{t_{1}}^{t-T}a_{R}\left(\tau \right)d\tau dt$.

At the any moment of t during a lane change, $S_{d}\left(t\right)$ can be calculated as:

(4) $$S_{d}\left(t\right)=S_{d}\left(t_{1}\right)+\left[S_{S}\left(t\right)-S_{R}\left(t\right)\right]\;t\in \left[t_{1},t_{2}\right]$$

where $S_{d}\left(t_{1}\right)$ is the relative longitudinal distance between S and R at *t*_1_.

At t_2_, if the relative velocity $V_{r}\left(t_{2}\right)$ is close to or lower than 0 m/s, even if the distance between S and R is small, the TTC will be small, and the moment is considered a safe stage [21]. However, drivers tend to keep a minimum safe distance ($D_{t2}$) at t_2_. Therefore, $S_{d}\left(t\right)$ at *t*_2_ can be calculated as:

(5) $$S_{d}\left(t_{2}\right)=S_{d}\left(t_{1}\right)-V_{r}\left(t_{1}\right)t_{2}+\frac{1}{2}a_{R}{\left(t_{2}-T\right)}^{2}-D_{t2}\geq 0\;V_{r}\left(t_{2}\right)\leq 0$$

(6) $$V_{r}\left(t_{2}\right)=V_{R}-a_{R}\left(t_{2}-T\right)-V_{S}$$

Furthermore, if $V_{r}\left(t_{2}\right)$ is higher than 0 m/s, to avoid a collision with S, the rear vehicle driver will maintain the previous or a greater deceleration until ***V_r_*** is equal to 0 m/s. If $V_{r}\left(t_{2}\right)$ is higher than 0 m/s, $S_{d}\left(t_{2}\right)$ should be higher than a safe distance ($D_{S}$), the $D_{S}$ can help ensures safety before the relative speed is reduced to 0 m/s, the $D_{S}$ can be calculated as:

(7) $$D_{S}=\frac{V_{r}^{2}\left(t_{2}\right)}{2a_{R}}\leq S_{d}\left(t_{2}\right)V_{r}\left(t_{2}\right)\leq 0$$

Therefore, $S_{d}\left(t_{2}\right)$ can be calculated as:

(8) $$S_{d}\left(t_{2}\right)=S_{d}\left(t_{1}\right)-\frac{V_{r}^{2}\left(t_{1}\right)}{2a_{R}}-V_{r}\left(t_{1}\right)T-D_{t2}\geq 0\;V_{r}\left(t_{2}\right)\leq 0$$

In addition, if the relative speed is small, a lane change under these conditions is theoretically safe. However, the driver will consider the lane change unsafe when the relative distance at *t*_1_ is small. That is, the lane-change decision model will only perform the lane change if the $S_{d}\left(t_{1}\right)$ is greater than a minimum acceptance distance ($D_{t1}$). Therefore, to perform a safe lanechange, the following two conditions must be met:

(9) $$\{\begin{array}{c} S_{d}\left(t_{1}\right)\geq D_{t1}\\ S_{d}\left(t_{2}\right)\geq 0 \end{array}$$

$S_{d}\left(t_{1}\right)$ and $V_{r}\left(t_{1}\right)$ are collected using the millimeter wave radar; t_2_, $D_{t1}$, and $D_{t2}$ are analyzed based on the naturalistic driving data, and $a_{R}$ is calculated using Equations (5) and (8).

### 4.3. Parameter Calibration

**Figure 7:**
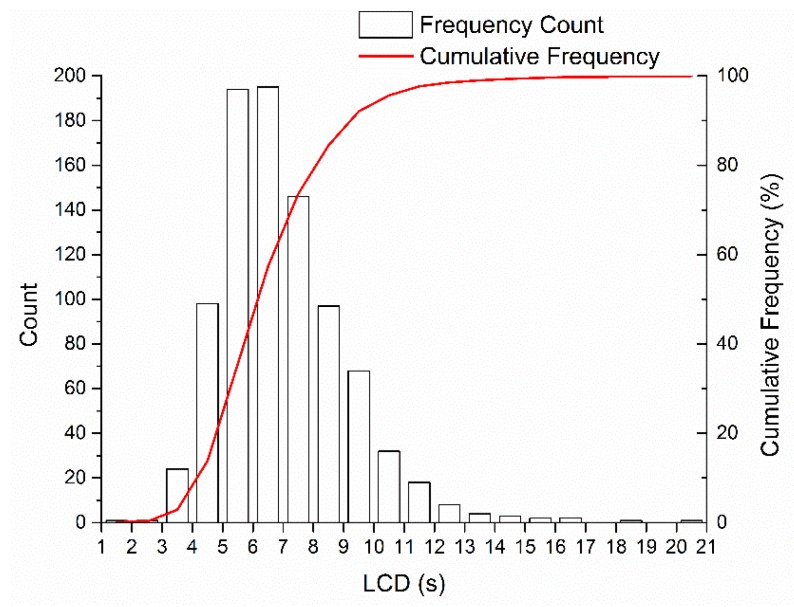
Lane-Change Duration (LCD) distribution.

During the naturalistic lane-change trial, 895 lane-change processes were recorded; 317 of which had R approaching quickly. A statistical analysis on all the lane-change processes was conducted and the LCD distribution is shown in Figure 7. The LCD ranged from 1.6 to 20.0 s, with a median of 6.6 s, a mean of 7.0 s, and a standard deviation of 2.1 s. After the lane-change start moment, some drivers slowly changed lanes, waiting to be overtaken by R, which resulted in the LCDs of this experiment being higher than LCDs recorded in the previous studies [48,49].

Based on the definition of TLC, this paper calculated TLC using the distance between the vehicle’s front wheel and the lane mark collected by the lane mark recognition system (Mobileye C2-170).

**Figure 8:**
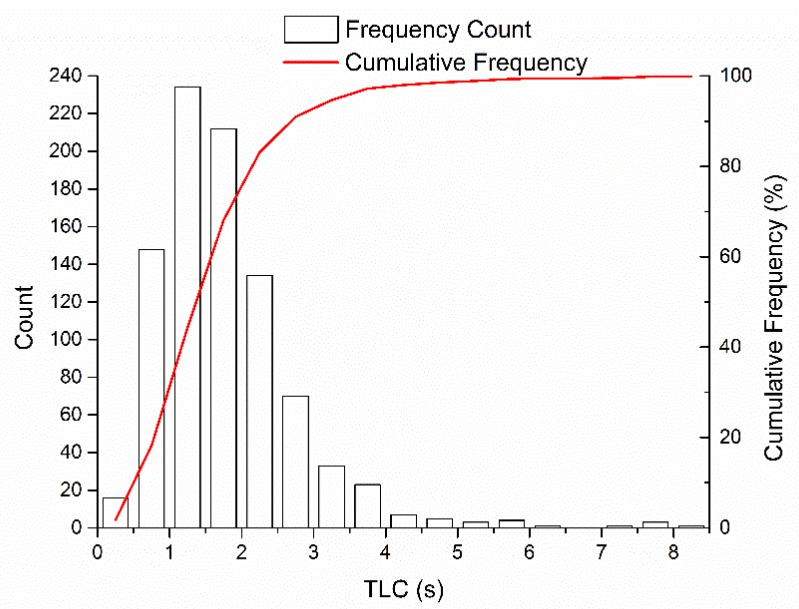
Time to Line Crossing (TLC) distribution.

The TLC distribution is shown in Figure 8. The TLC ranged from 0.2 to 8.3 s, with a median of 1.6 s, a mean of 1.7 s, and a standard deviation of 1.0 s. A total of 2.8% of the TLC during the lane-change processes were > 4 s, the result of some drivers slowly changing lanes to allow R to overtake, which helps avoid a collision with R. Furthermore, 85.8% of the TLC ranged between 0.2 and 2.5 s. In this model, we calibrated the TLC as 1.6 s.

**Figure 9:**
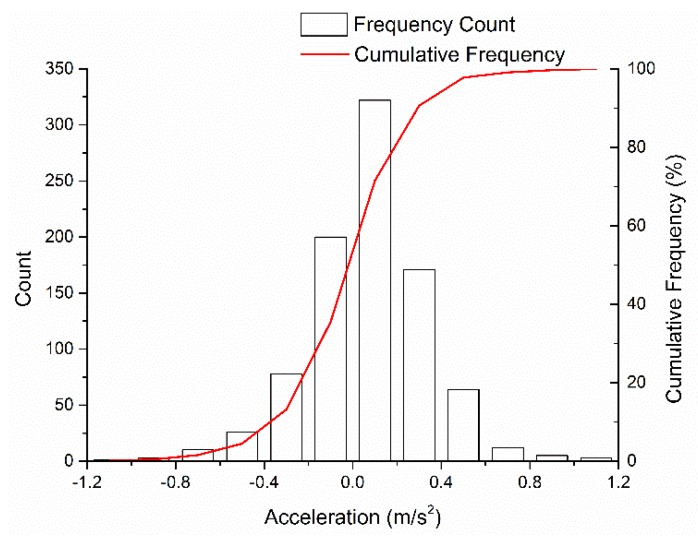
Acceleration distribution.

The distribution of the test vehicle acceleration during the lane changes is shown in Figure 9. Acceleration ranged from –1.02 to 1.16 m/s^2^, with a median of 0.06 m/s^2^, a mean of 0.07 m/s^2^, and a standard deviation of 0.27 m/s^2^. The acceleration was close to 0 m/s^2^, which implies that the driving speed is maintained during the lane change, which confirms the previous study [43].

### 4.4. Minimun Acceptance Distance (D_t1_) and Minimum Safe Distance (D_t2_)

On the highway, a driver may not perform a lane change when the relative distance at *t*_1_ is small. To investigate $D_{t1}$, we collected the relative distance before *t*_2_ from the data set of successful lane changes. The results showed a maximum is 204.60 m, a minimum is 4.59 m, a mean of 42.33 m, and a standard deviation of 32.51 m. Therefore, $D_{t1}$ was determined as 4.59 m.

After *t*_2_, even when the relative speed is close to 0 m/s, the front and rear drivers try to maintain a suitable safe distance. Sultan et al. [50] suggested that the relative speed is considered low when in the range of [–1.5 m/s, 1.5m/s]. To determine $D_{t2}$, we collected the relative distance when relative speed after *t*_2_ was low. The results showed a maximum of 175.20 m, a minimum of 3.25, a mean of 32.12 m, and standard deviation of 27.60 m. Therefore, $D_{t2}$ was determined as 3.25 m.

### 4.5. Extreme Moment Data

**Figure 10:**
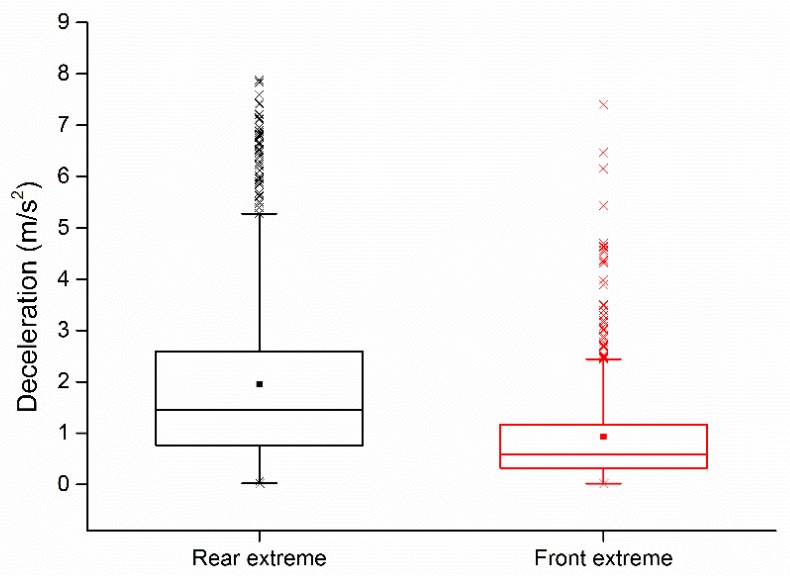
Minimum Safe Deceleration (MSD) for the extreme moment.

In the extreme moment trials, to ensure the safety of the driver, lane changes were not performed during the experiment. Participants were instructed only to push the wireless button to indicate the extreme moment. Based on the relative longitudinal distance and relative speed between R and T (F and T) at the subjective extreme moment, the MSD of R was calculated using the proposed lane-change decision model in Section 4.2. The MSDs at the front extreme moment and the rear extreme moment from different participants are shown in Figure 10.

In the rear extreme moment trial, 1300 rear extreme moments were collected, and the corresponding MSDs of R were calculated using the lane-change decision model. As shown in Figure 10, the decelerations ranged from 0.02 to 7.88 m/s^2^; the 25th, median, and 75th percentiles were 0.75 m/s^2^, 1.42 m/s^2^, and 2.58 m/s^2^, respectively; the mean was 1.95 m/s^2^, and the standard deviation was 1.66 m/s^2^. The result showed that from the perspective of the participants in the rear extreme trial, half of the participants in the rear extreme trial felt they could safely change lanes while the MSD is higher than 1.42 m/s^2^, and 75% of participants in the rear extreme trial cannot accept a MSD that is more than 2.58 m/s^2^.

Based on the statistical analysis of the rear extreme moment trial results, the second safe threshold (ST_2_) was initially set between the 75th percentile and the median, namely 1.42 to 2.58 m/s^2^.

In the front extreme moment trial, 912 front extreme moments were collected, and the corresponding MSDs of R were calculated using the lane-change decision model. As shown in Figure 10, the decelerations ranged from 0.02 to 7.40 m/s^2^; the 25th, median, and 75th percentiles were 0.29 m/s^2^, 0.50 m/s^2^, and 1.12 m/s^2^, respectively; the mean was 0.93 m/s^2^, and the standard deviation was 1.01 m/s^2^. The results showed that the subjective MSD of half the participants in the front extreme trial was higher than 0.50 m/s^2^, and 75% of the participants in the front extreme trial cannot accept a MSD of more than 1.12 m/s^2^, as it likely triggers a high level of anxiety in the drivers [51]. 

Based on the statistical analysis of the front extreme moment trial results, the primary safe threshold (ST_1_) was initially set between the 75^th^ percentile and the median, namely 0.50 to 1.12 m/s^2^.

The *t*-test was used to compare the subjective perception of the drivers in the front and rear extreme trial on lane-change safety. The Levene’s Test for equality of variances results showed that F(912, 1300) = 216.515, *p* < 0.001, which means the variances among the two driver types is not equal. The Welch’s *t*-test for equality of means results shown that *t*(2117.681) = 26.619, *p* < 0.001. The subjective perception of lane-change safety between participants in the rear extreme trial and participants in the front extreme trial have significant differences; in particular, the required deceleration in the rear extreme trial was higher than that of the front extreme trial. These results indicate that during the lane change, the participants in the front extreme trial were more aggressive, and the required deceleration safety level was higher in the front extreme trial than that in the rear extreme.

## 5. Threshold Determination

### 5.1. Signal Detection Theory

In order to establish the two-level safe lane-change thresholds that consider the driver’s risk perception in different trials, we used the SDT, which is widely applied in the determination of the optimal threshold for human perception [52,53]. 

Signals and noise have different definitions among various psychology fields, and SDT was used to discriminate between them. Before performing the lane-change maneuver, the signal and noise was defined as a safe and unsafe signal. When the MSD is lower than the safe threshold, the lane-change decision system permits the lane change to be executed; if not, then the system decides to wait for the proper time to perform the lane change. In the natural driving experiment, the lane-change data was used to verify the safe threshold. The safety lane-change process in the natural driving experiment was defined as the safe lane change, and the data of the failed lane change was defined as the unsafe lane change. The lane-change decision matrix is shown in Table 1.

Performing a lane change under the safe condition is correct and is termed Hit; performing a lane change in the unsafe condition is incorrect and is defined as a False alarm. When the lane-change decision system neglects the safe lane-change situation, it is incorrect and is termed as a False negative; waiting in the unsafe lane-change situation is correct and is defined as a Correct rejection. Both Hit and Correct rejection are correct signals, and the correct rate is termed the accuracy (*P_A_*). In this paper, the *P_A_*, False negative rate (*P_FA_*), and False alarm rate (*P_FN_*) were used to evaluate the decision model’s performance. The same *P_FN_*, a higher *P_A_*, and lower *P_FA_* indicate the better performance by the decision model.

The accuracy is calculated as:

(10) $$P_{A}=1-\frac{N_{FA}+N_{FN}}{N_{S}+N_{U}}$$

The False alarm rate is calculated as:

(11) $$P_{FA}=\frac{N_{FA}}{N_{S}}$$

The False negative rate is calculated as:

(12) $$P_{FN}=\frac{N_{FN}}{N_{U}}$$

where the *N_S_* is the total number of safe lane changes, *N_U_* is the total number of unsafe lane changes, N_FN_ is the number of False negatives, and *N_FA_* is the number of False alarms.

We selected the optimal safe thresholds within the range of ST_1_ and ST_2_ obtained in the previous section. The ST_1_ ranged from 0.50 to 1.12 m/s^2^, and the ST_2_ ranged from 1.42 to 2.58 m/s^2^. To calculate *P_A_*, *P_FA_*, and *P_FN_* at different MSD, the 0.01 m/s^2^ was selected as the step length. Two level safe thresholds were determined by considering the *P_A_*, *P_FA_*, and *P_FN_*.

### 5.2. Primary Safe Threshold (ST_1_) Selection

In our two-level lane-change decision model, the emphasis on the two safe thresholds are different. For the ST_1_, the major aim is to ensure that the S lane-change maneuver will not have a serious negative impact on the rear vehicle driver’s driving behavior, e.g., accelerating to avoid being cut-in, emergency braking, or anxiety and/or road rage. Therefore the primary deceleration threshold was selected within 0.50 to 1.12 m/s^2^, which meets most rear vehicle drivers’ expectations for safe car-following after cut-in events.

**Figure 11:**
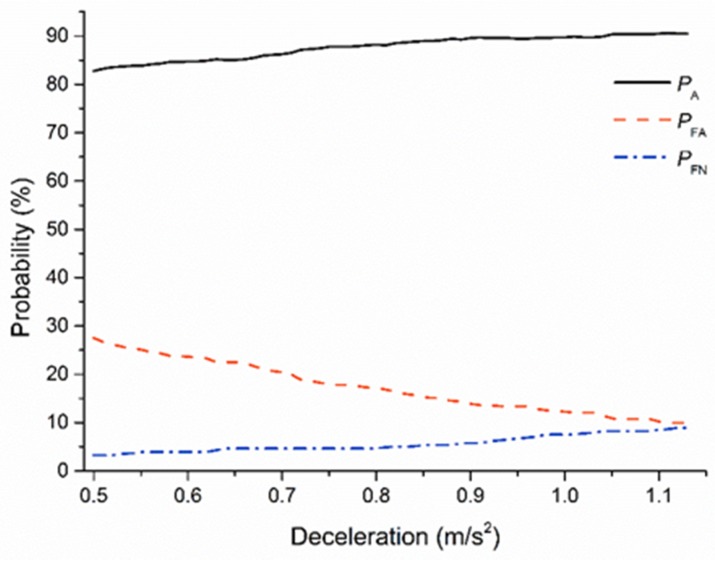
Signal Detection Theory (SDT) for the primary safe threshold (ST_1_).

*P_A_*, *P_FA_*, and *P_FN_*, at different ST_1_ are shown in Figure 11. The results showed that *P_A_* and *P_FN_* increase in parallel with MSD, while *P_FA_* decreases as a function of increasing MSD. The purpose of ST_1_ in the proposed model is to minimize the effect of S lane-change behavior on the R driver, and to avoid all potential dangerous situations to the highest extent possible. The higher *P_FN_* may trigger to the more potential risk. Therefore, the major aim of ST_1_ is to reduce the *P_FN_*. Within the range of ST_1_, all the *P_FN_* ranged from 3.2% to 8.9%; the *P_FN_* was lower than 5% when the safe threshold was less than 0.85 m/s^2^. 

In addition, the higher the threshold, the higher the *P_A_* and the lower the *P_FA_*. This trend indicates better performance of the lane-change decision system. When the threshold was 0.85 m/s^2^, *P_A_* and *P_FA_* reached 88.8% and 15.7%, respectively.

Therefore, by considering *P_A_*, *P_FA_*, and *P_FN_*, ST_1_ was determined as 0.85 m/s^2^; while *P_A_*, *P_FA_*, and *P_FN_* were 88.8%, 15.7%, and 5.0%, respectively. The primary safety threshold minimizes the occurrence of potential risks while ensuring accuracy, and satisfies the expectations of more than half of the rear vehicle drivers for safe car-following.

### 5.3. Secondary Threshold (ST_2_) Selection

Unlike ST_1_, which focuses on rear vehicle drivers and all the associated potential risks, the main target of ST_2_ is the subject vehicle-driver’s risk perception and ensuring the decision system reliability. Whether the safe threshold is higher or lower than the driver’s perceived safe acceleration, it still could possibly reduce the driver’s acceptance and trust of the intelligent assistance system. Therefore, ST_2_ was selected by considering the risk assessment of the subject vehicle driver in the rear extreme trial, which ranged from 1.42 m/s^2^ to 2.58 m/s^2^.

**Figure 12:**
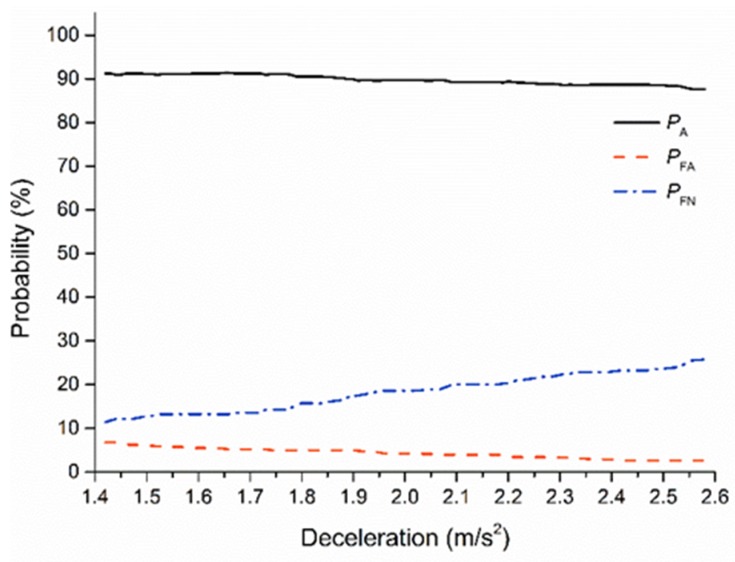
SDT for the second safe threshold (ST_2_).

*P_A_*, *P_FA_*, and *P_FN_* at different ST_2_ are shown in Figure 12. Within the range of ST_2_, changes of ST_2_ had little effect on *P_A_*, which was generally stable at around 90%. *P_FN_* rapidly increased with the increase of ST_2_, and *P_FA_* was lower than 7%. According to the purpose of the ST_2_ in the proposed model, the major aim is to increase the decision system accuracy, which could improve the acceptance and trust in the lane-change decision system. Thus, the *P_A_* needs to be considered first while selecting the secondary safe threshold. 

Within the range of the secondary safe threshold, *P_A_* fluctuated between 87.6% and 91.4%. When ST_2_ was less than 1.87 m/s^2^, the *P_A_* exceeded 90%.

In addition, *P_FA_* and *P_FN_* is an important factor for the improvement of the driver trust and acceptance. False alarm denotes performing a lane change in unsafe condition, which may reduce the trust, and false negative denotes waiting in a safe condition, which may reduce the acceptance. Thus, the *P_FA_* and *P_FN_* were taken into consideration while determining ST_2_. When the safe threshold was higher than 1.76 m/s^2^, the growth rate of *P_FN_* increased. In addition, the *P_FA_* was less than 5.0% when the threshold was 1.76 m/s^2^. 

Therefore, by considering *P_A_*, *P_FA_*, and *P_FN_*, ST_2_ was determined as 1.76 m/s^2^; *P_A_*, *P_FA_*, and *P_FN_* were 91.1%, 4.9%, and 14.3%, respectively. ST_2_ can improve the trust and acceptance of the subject vehicle driver in the lane-change decision system.

### 5.4. Summary of the Lane-Change Decision Model

According to the two-level safe thresholds, the decision rule is:

(13) $$Decision=\left\{ \begin{array}{ll} \mathrm{Safe}\;\mathrm{and}\;\mathrm{polite} & \mathrm{if}\;\mathrm{MSD}\leq {\mathrm{ST}}_{1}\\ \mathrm{Safe}\;\mathrm{but}\;\mathrm{impolite} & {\mathrm{if}\;\mathrm{ST}}_{1}<\mathrm{MSD}\leq {\mathrm{ST}}_{2}\\ \mathrm{Waiting} & \mathrm{if}\;\mathrm{MSD}>{\mathrm{ST}}_{2} \end{array}\right.$$

In the decision model, “Safe and good” denotes an opportunity in which AVs can perform a safe and polite lane change, which will not negatively affect the rear vehicle. “Safe but impolite” means that the AVs can safely execute a lane change, but this maneuver may disturb the driving behavior of the rear vehicle. “Waiting” means a rear-end collision may occur if AVs change lane at this moment.

To verify the lane-change decision model’s safety, the ISO model was compared with the decision model. ISO [21] considered TTC as the indictor and proposed a multi-level lane-change safe threshold for different relative speeds while R was quickly approaching S. It suggested that the TTC threshold was 2.5 s when the relative speed less than 10 m/s; the TTC threshold was 3.0 s when the relative speed ranged from 10 to 15 m/s; and the TTC threshold was 3.5 s when the relative speed ranged from 15 to 20 m/s.

**Figure 13:**
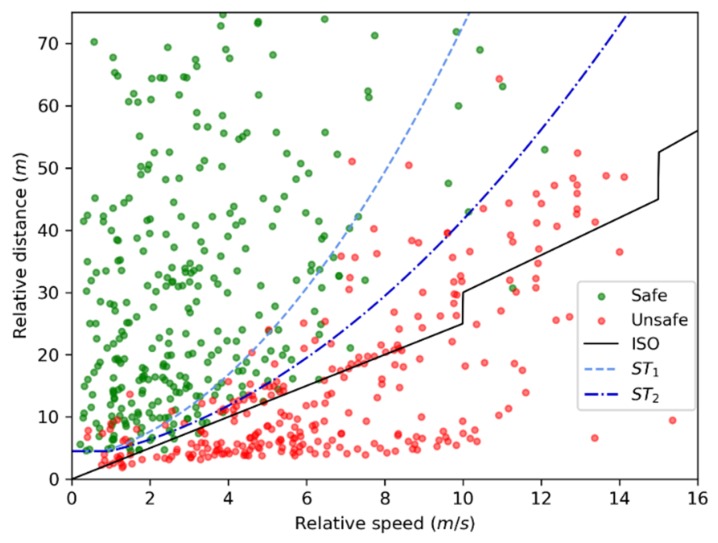
The comparison between the International Standards Organization (ISO) model and lane-change decision model.

The dataset of safe lane changes and unsafe lane changes was used to evaluate the lane-change decision model’s performance and the ISO model. The result is shown in Figure 13.

A summary of *P_A_*, *P_FA_*, and *P_FN_* of the ISO model and the proposed model are shown in Table 2. Compared with the ISO model, *P_A_* for both ST_1_ and ST_2_ was higher. Although *P_FA_* of the ISO model is small, *P_FN_* of the ISO model is far higher than that of lane-change decision model. The result indicates that the lane-change decision model has high safety reliability.

## 6. Discussion and Conclusions

In this work, we established a lane-change decision model with a two-level safe threshold in mixed traffic, which considers the rear vehicle’s deceleration behavior. The parameters in this model were calibrated based on the naturalistic lane-change behaviors. The two-level safe thresholds were determined according to the driver’s various risk perceptions—as the driver of R and driver of F—which were investigated in the front and rear extreme trial. The decision model was evaluated using SDT, which takes into account various risk perception of different drivers and real lane-change data. ST_1_ and ST_2_ were determined as 0.85 m/s^2^ and 1.76 m/s^2^, respectively.

The rear vehicle’s MSD was selected as an indicator to evaluate the lane-change safety, which was different from other widely used indicators, such as TTC, Time Headway (THW) (the ratio of relative distance between subject vehicle and front vehicle to the speed of subject vehicle), and TTCi (the reciprocal of TTC value) [54,55,56]. The MSD is an intuitive indicator, which is directly related to the maneuverability and willingness of the rear vehicle driver. A progressively higher MSD means the rear vehicle driver has to make a faster and greater response, which may increase the occurrence of the rear-end collision.

Two different extreme moment experiments were conducted on a highway to examine the lane-change behavior and risk perception of the driver. In the experiments, a vehicle outfitted with instruments was used to collect the information of surrounding vehicles and the subject vehicle’s driving data.

The lane change behavior was investigated based on the naturalistic lane-change experiment. The lane-change behavior measures assessed for the decision model were: LCD, TLC, and deceleration. Compared with the previous studies [47,48,57], both LCD and TLC were slightly higher, for two primary reasons. This experiment was carried out on the highway, a more dangerous environment for a vehicle to be fast approaching the test vehicle. Some drivers tended to slowly change lanes and let the rear vehicle overtake them to avoid collisions. Moreover, some researchers [58,59,60], found that the vehicle and driving environment model effect the driving behavior. Thus, the test vehicle and driving route model in this study may impact the LCD and TLC. Furthermore, the acceleration behavior during the lane change was investigated. The results validated those of a previous study [40], which reported that the driving speed is kept constant during the lane change. Therefore, we simplified the lane-change decision model by ignoring the car’s speed change during the lane-change process. 

The various risk perceptions of the rear vehicle driver and the front vehicle driver were explored using front and rear extreme trials. The MSD was used to characterize the driver’s risk perception, which was calculated using the proposed model. Compared to the driver of the preceding car, when the participant is a driver of the rear car, the perceived MSD is significantly smaller, which indicates that the rear car driver is more cautious during the lane-change process. Using SDT, the two-level thresholds were determined by considering the different perceived MSD in the two extreme trials. 

The major benefit of this study is the determination of two safe thresholds, each of which has a different role. The rear vehicle driver’s safety and risk perception is primarily accounted for by ST_1_, which was examined in the front extreme trial. The lane-change process can be viewed as the game process between the front vehicle and the rear vehicle [61], in which the preceding vehicle’s lane-change behavior greatly affects the rear vehicle driver’s driving behavior and emotion [62,63]. This implies that dangerous lane-change behavior not only leads to a potential rear-end collision between the front and rear vehicles, but also triggers the rear vehicle driver to engage in dangerous driving behavior, which increases the likelihood of the rear car colliding with other surrounding vehicles. Therefore, determination of ST_1_ based on the rear vehicle driver’s risk perception alleviates the driver’s anxiety and improves the rear vehicle’s safety.

The proposed ST_1_ was determined based on the rear extreme trial results analysis. Compared with the existing lane-change decision system, the secondary safe threshold not only ensures lane-change safety, but also accounts for the driver’s expectation, which minimizes the decision model interference on the driving behavior. ST_1_, which is established by the driver’s subjective perception, enhances the driver’s acceptance and trust of the intelligent driving system and contributes to the intelligent assistant system popularization [33,64].

Although this model’s thresholds were based on the subjective feelings of different drivers, the accuracy was guaranteed. Compared with the ISO model, this model’s accuracy was higher at both two-level thresholds, which indicates that this model improves lane-change safety, while ensuring the driver’s comfort.

The decision model can be used in different driving situations. In the free condition [9], AVs can perform a safe and polite lane-change maneuver. In the force condition or in a hurry, AVs can perform the safe but not polite lane-change maneuver, which can save passengers time on the basis of safety.

The novel human-like lane-change decision model can find a more suitable time to change lanes in mixed traffic, which ensures the subject vehicle’s safety and reduces its interferences with the rear vehicle, thus further ensuring safety all around.

A few aspects of our work need to be improved in future research. Different driving styles may reflect different subjective perceptions of the ability to safely change lanes. Assuming that the numbers of samples are adequate, future study will focus on establishing diverse thresholds on the basis of different driving styles to enhance the acceptability of the proposed lane-change decision model. In addition, model parameters need to be calibrated based on a more sufficient number of samples.

## Figures and Tables

**Table 1:** The lane-change decision matrix.

	Safe Lane Change	Unsafe Lane Change
Safe signal	Hit	False alarm
Unsafe signal	False negative	Correct rejection

**Table 2:** The lane-change decision matrix.

	*P_A_* (%)	*P_FA_* (%)	*P_FN_* (%)
ST_1_	88.8	15.7	5.0
ST_2_	91.1	4.9	14.3
ISO model	82.0	1.3	40.1
